# Reducing overconfident errors in molecular property classification using Posterior Network

**DOI:** 10.1016/j.patter.2024.100991

**Published:** 2024-05-08

**Authors:** Zhehuan Fan, Jie Yu, Xiang Zhang, Yijie Chen, Shihui Sun, Yuanyuan Zhang, Mingan Chen, Fu Xiao, Wenyong Wu, Xutong Li, Mingyue Zheng, Xiaomin Luo, Dingyan Wang

**Affiliations:** 1 Drug Discovery and Design Center, State Key Laboratory of Drug Research, Shanghai Institute of Materia Medica, Chinese Academy of Sciences, 555 Zuchongzhi Road, Shanghai 201203, China; 2 University of Chinese Academy of Sciences, 19A Yuquan Road, Beijing 100049, China; 3 School of Chinese Materia Medica, Nanjing University of Chinese Medicine, Nanjing 210023, China; 4 School of Physical Science and Technology, ShanghaiTech University, Shanghai 201210, China; 5 Lingang Laboratory, Shanghai 200031, China

**Keywords:** Uncertainty quantification, artificial intelligence-aided drug design, QSAR, trustworthy AI

## Abstract

Deep-learning-based classification models are increasingly used for predicting molecular properties in drug development. However, traditional classification models using the Softmax function often give overconfident mispredictions for out-of-distribution samples, highlighting a critical lack of accurate uncertainty estimation. Such limitations can result in substantial costs and should be avoided during drug development. Inspired by advances in evidential deep learning and Posterior Network, we replaced the Softmax function with a normalizing flow to enhance the uncertainty estimation ability of the model in molecular property classification. The proposed strategy was evaluated across diverse scenarios, including simulated experiments based on a synthetic dataset, ADMET predictions, and ligand-based virtual screening. The results demonstrate that compared with the vanilla model, the proposed strategy effectively alleviates the problem of giving overconfident but incorrect predictions. Our findings support the promising application of evidential deep learning in drug development and offer a valuable framework for further research.

## Highlights

• AttFpPost uses normalizing flow to enhance the uncertainty estimation capability
• AttFpPost can alleviate OF predictions on ADMET tasks
• We construct a SOTA model based on AttFpPost for classifying P-gp inhibitors
• AttFpPost shows enhanced early enrichment capability in LBVS

## The bigger picture

Artificial-intelligence-driven deep-learning models have been widely used in drug design, but traditional models may produce incorrect results for samples that fall outside the coverage of the original training datasets. This can lead to an influx of unreliable prediction results into subsequent drug development pipelines. A new model is proposed here that is better able to estimate uncertainty for predictions outside the original training domain, helping reduce overconfident but incorrect predictions.

## Teaser

This study investigates the use of deep-learning models for predicting molecular properties in drug development, addressing the significant issue of overconfident predictions for out-of-distribution samples by traditional models. By integrating normalizing flow in place of the Softmax function, overconfident mispredictions are reduced. Validated across various scenarios, these findings highlight the potential of evidential deep learning to improve the accuracy and efficiency of drug development.

## Introduction

With the rapid development of artificial-intelligence (AI)-driven drug design (AIDD), deep-learning (DL) models have shown great success in improving the efficiency and success rate of the drug development pipeline.^1^^,^^2^^,^^3^^,^^4^^,^^5^^,^^6^ It has been widely acknowledged that avoiding the introduction of highly unreliable predictions into the decision-making process is particularly crucial for drug discovery.^7^ Thus, within the various research domains of AIDD, uncertainty estimation has gained considerable attention in recent years.^8^^,^^9^^,^^10^^,^^11^^,^^12^^,^^13^^,^^14^

Uncertainty estimation assesses the level of confidence in the predictions made by the model, aiming to prevent unreliable prediction results from influencing real decision-making processes.^15^ The inconsistency between DL models and actual human behavior patterns is one of the motivations behind uncertainty estimation research.^16^ Unlike humans, who can provide a measure of confidence when responding to questions, such as confidently giving answers within their knowledge domain or honestly admitting “I don’t know,” traditional DL models often struggle to provide (or accurately provide) confidence in their predictions.^17^ Indeed, neural networks are capable of providing a prediction value $\hat{y}$ for any given input. However, this limitation can lead to the influx of unreliable prediction results into subsequent decision pipelines, resulting in significant wastage of resources.

Currently, the relevant research on uncertainty estimation in AI-based drug design primarily focuses on regression problems, with the main objective of providing uncertainty estimation or prediction intervals for the predicted values.^18^^,^^19^^,^^20^^,^^21^^,^^22^^,^^23^ Compared to regression problems, the research on uncertainty estimation in classification problems within the field of drug design is relatively limited, which is primarily attributed to the general assumption that for binary classification problems (the scenario discussed in this study does not involve multi-classification problems), the predicted probability p, a number ranging from 0 to 1 that represents the probability of the sample belonging to the positive class, inherently contains implicit uncertainty information. For example, if p is 0.99, it indicates that the model is highly certain that the sample is positive. Conversely, a p value of 0.5 indicates that the model is uncertain. In practical applications, researchers tend to select samples with high confidence, such as those with p greater than 0.9 or samples ranked at the top, for subsequent experimental validation. This requirement implies that high-confidence samples provided by the model should indeed exhibit higher reliability. However, previous research has noted three defects associated with the predicted probabilities p generated by traditional neural networks based on the Softmax function (and similarly with the Sigmoid function, as they are essentially the same in binary classification problems):

(1) For out-of-distribution (OoD) samples, which are samples that fall outside the coverage of the training dataset and the application domain, the predicted probabilities p do not exhibit the expected distribution around high uncertainty at 0.5.^24^ Instead, they may give predictions that fall within the high-confidence range (such as being less than 0.1 or greater than 0.9). This behavior leads to the occurrence of “overconfident false” (OF) results, where the model exhibits high confidence in its predictions despite being incorrect.
(2) The calibration of p is often poor, precluding its use as an indicator of a reliable confidence level.^25^ For example, among all samples with a predicted probability p of 0.9, the actual proportion of positive samples may only be 70%.
(3) The only type of uncertainty that can be reliably captured by p is aleatoric uncertainty,^26^ which is related to the inherent noise of the input. However, it is inappropriate to capture epistemic uncertainty,^27^ which is related to the lack of sufficient training data. However, in some scenarios such as active learning, the guidance of epistemic uncertainty is essential.^9^^,^^12^

**Figure 1:**
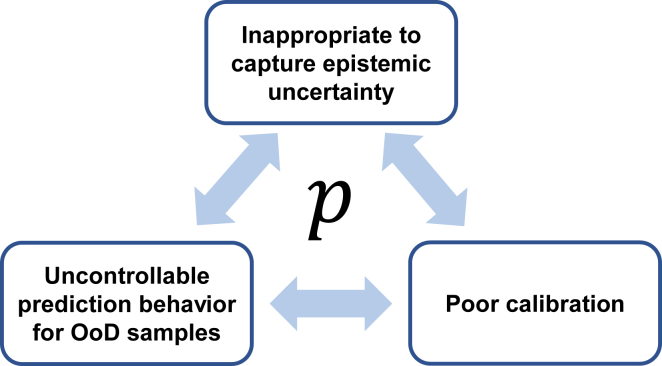
The deficiencies of probability given by the Softmax-based deep-learning models

These three defects are not independent but rather interrelated (Figure 1). In the context of molecular property prediction using graph neural networks (GNNs), previous research has also demonstrated the presence of similar problems.^28^^,^^29^ In this study, we also illustrate the limitations of Softmax-based classification models using a specially designed synthetic dataset.

To solve the aforementioned problems, two categories of solutions have been proposed. The first approach is to calibrate the predicted p of the test set with reference to the p and label distribution of the validation set. Mervin et al.^25^ investigated the effects of three calibration methods on 2,112 drug targets using AstraZeneca’s internal dataset and found that the overconfidence of the model was significantly reduced after calibration. However, probability calibration can generally only adjust the probability distribution and not correct the ranking; i.e., it can solve problem (2), but not (1) and (3). The second approach is to use the Bayesian neural network (BNN) for modeling. Compared with the traditional modeling approach, the BNN has two major differences. First, in contrast to traditional neural networks that give a definite point prediction for each given input, the output given by the BNN exists in the form of a distribution. Second, BNNs employ Bayesian inference to obtain the posterior distribution of model parameters. Therefore, the parameters of a trained BNN model also exist in the form of probability distributions instead of fixed parameters. These two distinctions correspond to the aleatoric uncertainty and epistemic uncertainty associated with BNN predictions. Hwang et al.^28^ conducted a study on the effect of the BNN in the context of GNN-based QSAR classification models, demonstrating that the BNN can indeed obtain a classification model with improved calibration ability. However, even BNNs do not explicitly consider the distance in the feature space. Moreover, methods such as variational inference, which are employed to solve the posterior distribution, involve substantial computational overhead, making their deployment challenging in practical application scenarios.^30^^,^^31^

To solve the problem of high computational cost in training the BNN, a method called evidential DL (EDL) has emerged in recent years.^32^^,^^33^^,^^34^^,^^35^ The principle of EDL lies in the assumption that the distribution of multiple sets of predictions follows a higher-order distribution. By directly predicting the parameters of this higher-order distribution, EDL can obtain the estimation of model uncertainty, thus avoiding the need for time-consuming sampling or variational inference procedures. The rise of EDL has also captured the attention of cheminformatics researchers. Coley and coworkers found that EDL can obtain significantly improved uncertainty estimation results compared with traditional approaches in molecular property prediction.^14^ However, because the prior distribution assumed by EDL on regression problems cannot be directly applied to classification problems,^36^ the models proposed by Coley et al. cannot be directly adapted to the domain of classification problems.

In this study, we investigated the application of an EDL model in the context of molecular property prediction for classification tasks. We used the Posterior Network (PostNet)^37^ architecture to model the predicted probability distribution, replacing the Softmax function with a normalizing flow. Specifically, we took our previously developed Attentive Fp (AttFp) framework as the global feature extraction module and integrated it with PostNet to propose a model tailored for offering robust and reliable predictions in molecular property classification, named AttFpPost. In summary, our work makes the following main contributions:

(1) We conducted empirical studies to demonstrate that in both synthetic and real-world scenarios, AttFpPost can effectively alleviate the problem of giving OF predictions when faced with OoD samples in molecular property classification tasks, providing more reliable decision-making insights for drug development.
(2) We constructed a state-of-the-art model based on AttFpPost for classifying P-glycoprotein (P-gp) inhibitors.
(3) We demonstrated the improvement of AttFpPost on the screening power for the application scenario of ligand-based virtual screening (LBVS).

### Theoretical background

#### Estimate uncertainty by evidential deep learning

Taking the regression model as an example, the distribution of a prediction given by the BNN can be portrayed by a set of the mean μ and variance $\sigma^{2}$. The computational burden of the BNN arises from the difficulty of directly obtaining multiple sets of predictions ${\left\{\mu_{i},\sigma_{i}^{2}\right\}}_{i=1}^{n}$ given by the posterior distribution of the model, which requires the use of variational inference or Monte Carlo sampling.

EDL assumes that μ and $\sigma^{2}$ follow a higher-order distribution $p\left(\mu ,\sigma^{2}|m\right)$. By directly predicting the parameter m, EDL calculates the relevant statistics of μ and $\sigma^{2}$. For example, var[μ] represents epistemic uncertainty and $E\left[\sigma^{2}\right]$ represents the aleatoric uncertainty.^14^ Because a set of μ and $\sigma^{2}$ already represents a distribution, and $p\left(\mu ,\sigma^{2}|m\right)$ predicts the distribution of μ and $\sigma^{2}$, the evidence distribution is aptly referred to as “the distribution of distributions.”

#### Fundamentals of PostNet

For binary classification tasks, a categorical distribution $p^{\left(i\right)}=\left[1-p^{\left(i\right)},p^{\left(i\right)}\right]$ is commonly used to represent the predictive probability distribution ${\hat{y}}^{\left(i\right)}$ for the *i*-th sample:

(Equation 1) $$\begin{array}{c} {\hat{y}}^{\left(i\right)}∼\text{Cat}\left(p^{\left(i\right)}\right) \end{array}\text{,}$$

where Cat refers to categorical distribution. The problem with the Softmax function is that directly mapping the output $z^{\left(i\right)}$ from the last hidden layer to $p^{\left(i\right)}$ cannot guarantee that $z^{\left(i\right)}$ will be mapped to a high-uncertainty result (such as $p^{\left(i\right)}=0.5$) for OoD samples.

From a probabilistic standpoint, the output $p^{\left(i\right)}$ from traditional classification models lacks epistemic uncertainty primarily owing to its representation as a prediction from a single model. To estimate the epistemic uncertainty, obtaining the posterior distribution of $p^{\left(i\right)}$ is necessary. PostNet assumes that $p^{\left(i\right)}$ follows another distribution $q^{\left(i\right)}$:

(Equation 2) $$\begin{array}{c} p^{\left(i\right)}∼q^{\left(i\right)} \end{array}\text{.}$$

With this assumption, PostNet directly predicts the parameters of $q^{\left(i\right)}$ to obtain the posterior distribution of $p^{\left(i\right)}$, which represents the fundamental principle of EDL. Because the Dirichlet distribution is the conjugate prior for the categorical distribution in Bayesian statistics, PostNet assumes that $q^{\left(i\right)}$ follows a Dirichlet distribution to incorporate more prior information:

(Equation 3) $$\begin{array}{c} q^{\left(i\right)}=\text{Dir}\left(\alpha^{\left(i\right)}\right) \end{array}\text{,}$$

where Dir refers to the Dirichlet distribution, and $\alpha^{\left(i\right)}=\left[\alpha_{0}^{\left(i\right)},\alpha_{1}^{\left(i\right)}\right]$ represents the alpha parameters for the negative class $\left(\alpha_{0}^{\left(i\right)}\right)$ and the positive class $\left(\alpha_{1}^{\left(i\right)}\right)$. According to the nature of the Dirichlet distribution, the value of the parameter $\alpha_{c}^{\left(i\right)}$ can be interpreted as “confidence,” which is the probabilistic explanation for the inclusion of epistemic uncertainty in $\alpha_{c}^{\left(i\right)}$.

To obtain the estimation for $\alpha^{\left(i\right)}=\left[\alpha_{0}^{\left(i\right)},\alpha_{1}^{\left(i\right)}\right]$, PostNet uses probability densities in the latent space instead of the traditional Softmax function. Specifically, PostNet uses the probability density functions $P_{c}\left(z\right)$ from normalizing flow to calculate the probability densities of $z^{\left(i\right)}$ on the corresponding positions in the latent space.^38^ During the training process, the model maximizes the probability densities $P_{c}\left(z^{\left(i\right)}\right)$ on the regions corresponding to the samples of class c (c equals 0 or 1) in the latent space. For regions in the latent space where there are no training samples, $P_{c}\left(z\right)$ will maintain a low-probability prior distribution, close to 0. These two probability densities would undergo a specific transformation in Equation 4 to obtain $\alpha_{c}^{\left(i\right)}$, which can represent the “confidence” of the predictions of PostNet for the sample belonging to the negative or positive class:

(Equation 4) $$\begin{array}{c} \alpha_{c}^{\left(i\right)}=1+N_{c}P_{c}\left(z^{\left(i\right)}\right)\text{,} \end{array}$$

where $N_{c}$ is the number of samples belonging to class c in the training set.

According to Equation 4, a higher value of $\alpha_{c}^{\left(i\right)}$ can indicate that the model has a stronger confidence that the sample is negative (or positive). Simultaneously, PostNet can also provide predictive probability in the form of $\bar{p}$, which is similar to p:

(Equation 5) $$\begin{array}{c} {\bar{p}}^{\left(i\right)}=\frac{\alpha_{1}^{\left(i\right)}}{\alpha_{0}^{\left(i\right)}+\alpha_{1}^{\left(i\right)}}\text{.} \end{array}$$

When $P_{c}\left(z^{\left(i\right)}\right)$ is close to 0, $\alpha_{c}^{\left(i\right)}$ tends to be close to 1. This implies that for OoD samples, $\alpha_{0}^{\left(i\right)}$ and $\alpha_{1}^{\left(i\right)}$ are both close to 1. According to Equation 5, ${\bar{p}}^{\left(i\right)}$ will be close to a high-uncertainty value of 0.5. This ensures that the prediction for OoD samples remains controllable. Furthermore, according to the properties of the Dirichlet distribution, the expectation of $p^{\left(i\right)}$ in the posterior distribution of $p^{\left(i\right)}$ is exactly equal to the right-hand side of Equation 5:

(Equation 6) $$\begin{array}{c} E_{p^{\left(i\right)}∼\text{Dir}\left(\alpha^{\left(i\right)}\right)}\left[p^{\left(i\right)}\right]={\bar{p}}^{\left(i\right)}=\frac{\alpha_{1}^{\left(i\right)}}{\alpha_{0}^{\left(i\right)}+\alpha_{1}^{\left(i\right)}}\text{.} \end{array}$$

Moreover, since $\alpha_{c}^{\left(i\right)}$ is associated with $P_{c}\left(z^{\left(i\right)}\right)$, the value of $\alpha_{0}^{\left(i\right)}+\alpha_{1}^{\left(i\right)}$ can be used as an estimation of the epistemic uncertainty for a given sample. A smaller value indicates that the new sample is farther away from the latent space region defined by the training samples, resulting in higher epistemic uncertainty.

In summary, PostNet exhibits two key characteristics of interest. First, it provides “multiple-sets” predictions within one forward propagation, overcoming the computational burden associated with traditional BNN frameworks while retaining the advantages of BNN models. Second, for OoD samples, $P_{c}\left(z^{\left(i\right)}\right)$ tends toward infinitesimal values. According to Equations 4 and 5, $p^{\left(i\right)}$ will converge to 0.5. This avoids the problem of uncontrollable prediction behavior exhibited by traditional classification models when faced with OoD samples.

## Results

### Model architecture overview

**Figure 2:**
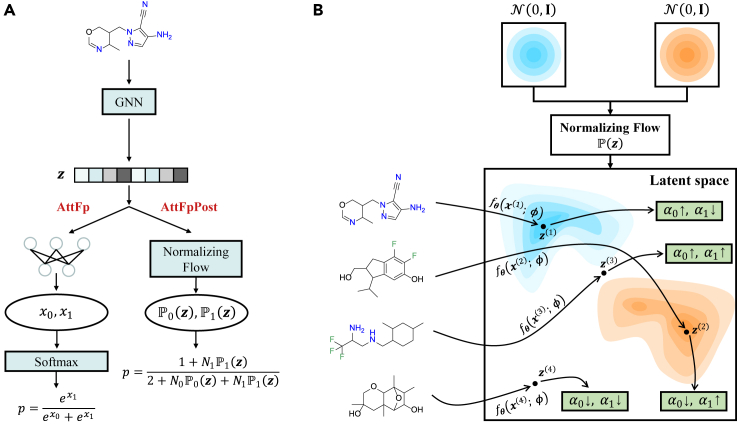
Illustration of PostNet (A) The difference between PostNet (AttFpPost) and the traditional classification network (AttFp). (B) The process of obtaining the PostNet output $\alpha^{\left(i\right)}$ by employing normalizing flow to the last hidden representation $z^{\left(i\right)}$ for four different molecules as inputs. From top to bottom, the four molecules represent the *Nmol* (negative sample), *Fmol* (positive sample), *N_Fmol* (sample with high aleatoric uncertainty), and *NULLmol* (sample with high epistemic uncertainty) used in the synthetic dataset experiment.

AttFp is a message-passing neural network model that incorporates attention mechanisms to capture important features for accurate molecular property prediction.^39^ Here, we adopt AttFp as the global feature extraction module and integrate it with PostNet to obtain AttFpPost. PostNet uses probability densities in the latent space to make classification instead of the traditional Softmax function (Figure 2A). Specifically, PostNet will initialize two trainable probability density functions $P_{c}\left(z\right)$ (c equals 0 or 1), corresponding to the negative class (0) and the positive class (1). Until the last hidden representation $z^{\left(i\right)}$ is obtained, there is no difference between PostNet and the traditional model. After obtaining $z^{\left(i\right)}$, $P_{c}\left(z\right)$ will return the probability densities $P_{c}\left(z^{\left(i\right)}\right)$ of the corresponding position in the latent space (Figure 2B). Finally, the model outputs the predictive probability p through Equations 4 and 5.

### Datasets

#### Synthetic dataset

Introducing noise or constructing specific data distributions in a synthetic dataset is a commonly used approach for analyzing the prediction behavior of DL models.^40^^,^^41^ To provide a more intuitive explanation of how PostNet decouples the overall uncertainty, we designed a synthetic dataset for experiments. The training set consists of three categories of molecules: *Nmol*—molecules containing element N (nitrogen) but lacking F (fluorine), labeled as negative; *Fmol*—molecules containing element F but lacking N, labeled as positive; and *N_Fmol*—molecules containing both elements N and F, randomly labeled as negative or positive with equal probability. The test set includes *Nmol*, *Fmol*, and *N_Fmol*, following the same labeling scheme as the training set. In addition, the test set contains a fourth category of molecules called *NULLmol*, which does not contain N or F elements. In this context, *NULLmol* can be explicitly considered as OoD samples. These four categories of molecules (*Nmol*, *Fmol*, *N_Fmol*, and *NULLmol*) are illustrated on the left side of Figure 2B. All molecules in the training and test sets are randomly selected from GDB-17.^42^

#### ADMET datasets

##### CardioTox

The CardioTox dataset was constructed by Han et al.^29^ specifically for studying the prediction behavior of molecular property classification models on OoD samples. The modeling task of the CardioTox dataset is to predict whether a molecule exhibits hERG toxicity. The data for this dataset were initially collected and curated by Siramshetty et al.^43^ for evaluating the performance of AI models in predicting molecular hERG toxicity. Han et al. adopted the training-test split method proposed by Siramshetty et al. and further divided the training set to create a validation set consisting of 1,631 molecules. According to Han et al., using this split method, 84% of the molecules in the test set are considered OoD samples. They defined OoD molecules as those with an average Tanimoto distance greater than 0.7 from the eight most similar molecules in the training set. This indicates that the test set exhibits significant OoD characteristics. We selected this dataset and followed the training-validation-test split method proposed by Han et al. to investigate the prediction behavior of PostNet on OoD samples.

##### Therapeutics Data Commons database

The Therapeutics Data Commons (TDC) database compiles and organizes nearly a hundred small-molecule datasets related to life sciences. It defines a fixed training-test split method (typically based on molecular scaffolds) for each modeling task to ensure fair comparisons between different models.^44^ To ensure the effective training of DL models, we have selected datasets with a minimum of 1,000 samples. Our selection includes the Pgp_Broccatelli,^45^ AMES,^46^ BBB_Martins,^47^ CYP3A4_Veith,^48^ and CYP2C9_Veith^48^ datasets, which will serve as benchmark datasets owing to their high relevance and significance in ADMET fields. For these five datasets, we used the training-test split method provided by TDC. In addition, we further randomly split the training set into five folds for 5-fold cross-validation and task-specific hyperparameter optimization.

The six different ADMET prediction tasks mentioned in "CardioTox" and "Therapeutics Data Commons database", along with their respective dataset names, total data size, numbers of positive and negative samples, modeling tasks, and dataset sources, are summarized in Table S1.

##### P-gp inhibitor external dataset

To further explore the predictive ability of PostNet on the ADMET tasks and facilitate a fair comparison with existing models, we collected a high-quality P-gp inhibitor external test set from the PharmaPendium database.^49^^,^^50^ We employed a rigorous data collection strategy and further conducted manual inspections based on the corresponding approval documents or literature provided by PharmaPendium. As a result, we obtained a total of 152 high-quality samples, among which 62 are positives and 90 are negatives, resulting in a relatively balanced distribution of positive and negative samples. More details of the data collection strategy are provided in the supplemental experimental procedures.

#### Ligand-based virtual screening dataset

The LIT-PCBA dataset^51^ is constructed based on high-throughput screening results from the PubChem database. It has been processed using computational methods to remove specific distribution biases, making it suitable for testing and comparing LBVS methods.^51^ Following the approach proposed by Shen et al.,^52^ we will select only targets with more than 100 positive samples in the training set for evaluation. The data profiles for each target are summarized in Table S2.

### Illustrating enhanced uncertainty estimation on a synthetic dataset

Due to the complicated nature of molecular structures, the definition of OoD for molecular property prediction is ambiguous.^53^ Previous studies typically identified OoD samples based on molecular scaffolds or molecular fingerprint distances.^29^^,^^54^ Thus, in this study, we designed a synthetic dataset comprising four categories of molecules and employed specific labeling and partitioning strategies to establish a clear definition of OoD under ideal conditions. The molecules in the test set exhibit varying degrees and types of uncertainties. For detailed construction procedures, please refer to “datasets”. It is important to emphasize that the synthetic dataset we constructed serves solely as an auxiliary tool to provide a controlled environment for illustrating the principles of AttFpPost. The task itself does not possess any practical application or real-world implications.

Evidently, the task on the designed synthetic dataset is effortless, as all *Nmol* and *Fmol* in the test set should be accurately classified. In contrast, both *N_Fmol* and *NULLmol* are unlikely to achieve predictions better than random. However, the reasons for the inability to predict these two categories of molecules are different. For *N_Fmol*, although there are a large number of similar molecules in the training set, their labels are assigned in equal proportions between negative and positive, making it impossible for the model to learn meaningful input-output relationships. For *NULLmol*, there are no similar molecules in the training set, and the lack of distributional information on labels for this category of molecules hinders the model’s ability to make accurate predictions. *N_Fmol* and *NULLmol* were designed to represent two distinct types of uncertainties, where the former is characterized by high aleatory uncertainty and low epistemic uncertainty, while the latter exhibits high epistemic uncertainty and low aleatory uncertainty.

**Figure 3:**
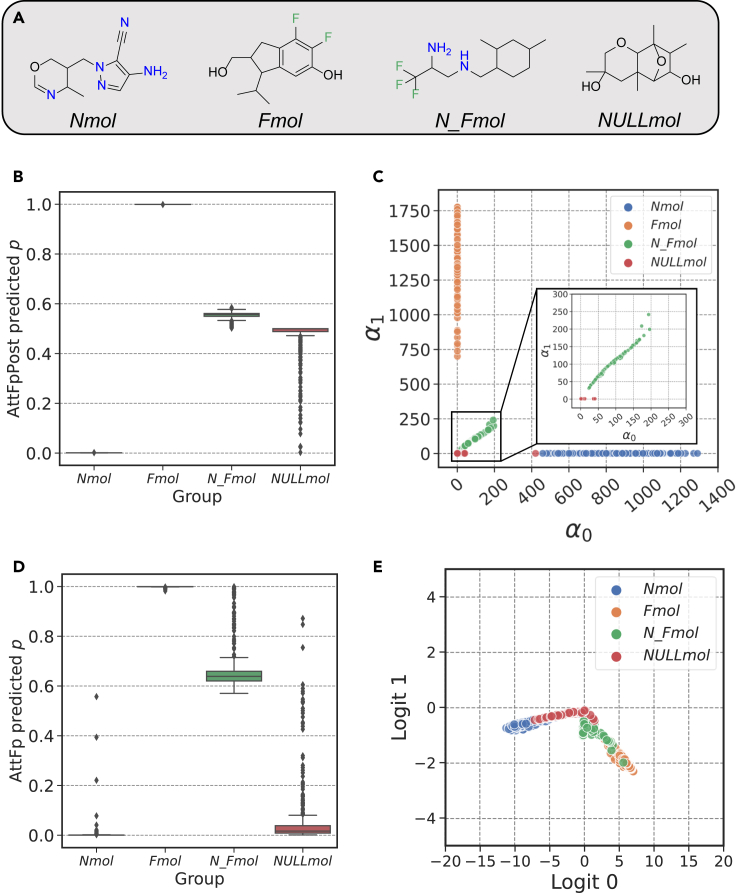
Comparison of prediction behavior between AttFp and AttFpPost on the synthetic dataset (A) Examples of *Nmol* (negative samples), *Fmol* (positive samples), *N_Fmol* (samples with high aleatoric uncertainty), and *NULLmol* (samples with high epistemic uncertainty). (B) The distribution of p given by AttFpPost on the test set. (C) The distribution of $\alpha_{0}$ and $\alpha_{1}$ given by AttFpPost on the test set. (D) The distribution of p given by AttFp on the test set. (E) The distribution of logit 0 and logit 1 given by AttFp on the test set.

Figure 3C shows the predictions of AttFpPost on the test set. In Figure 3C, each point represents a test sample, with the abscissa and ordinate representing the values of $\alpha_{0}$ and $\alpha_{1}$ given by AttFpPost, respectively. Figure 3B illustrates the distribution of probabilities p, transformed from Figure 3C using Equation 5. Figure 3D shows the distribution of the predicted probabilities p provided by AttFp with the traditional Softmax function on the test set. Figure 3E illustrates the logits of each test sample as provided by AttFp before passing through the Softmax function to obtain p.

From Figures 3B and 3D, it is evident that both AttFpPost and AttFp perform well in classifying *Nmol* and *Fmol*. Therefore, our focus is primarily on the prediction behavior of *N_Fmol* and *NULLmol*. From Figure 3D, it can be observed that AttFp predicts *N_Fmol* with p around 0.5, indicating a high level of uncertainty in the model’s predictions for *N_Fmol*, which is in line with expectations. However, for *NULLmol*, AttFp tends to give high-confidence negative predictions, which is contrary to expectations. In Figure 3E, the logits before Softmax fail to distinguish the four categories of molecules well, exhibiting significant overlap. These results confirm the two problems with traditional classification models mentioned in the introduction. First, classification models based on the Softmax function do not necessarily predict OoD samples around 0.5, as in this case, where the model incorrectly predicts high-confidence negative labels for *NULLmol* despite its inability to classify them. Second, even though AttFp’s predictions for *NULLmol* are close to 0.5, relying exclusively on the p or logits is insufficient to distinguish *NULLmol* from *N_Fmol*. This indicates that traditional classification models cannot differentiate the sources of uncertainty.

From Figure 3C, it is evident that the prediction distribution given by AttFpPost differs from that of Figure 3E, as AttFpPost effectively distinguishes between *N_Fmol* and *NULLmol*. The predictions on *N_Fmol* exhibit high values for both $\alpha_{0}$ and $\alpha_{1}$, since they possess characteristics of both positive and negative molecules. In contrast, *NULLmol* resembles neither negative nor positive, resulting in very small values for both $\alpha_{0}$ and $\alpha_{1}$, close to 1. This led to the gathering of all red points representing *NULLmol* in the bottom-left corner. Figure 3B illustrates that AttFpPost exhibited a distribution of p around 0.5 for both *N_Fmol* and *NULLmol*, indicating significant uncertainty.

In conclusion, this experiment has demonstrated two advantages of AttFpPost based on normalizing flow compared with traditional classification models. First, it provides a more accurate uncertainty estimation for OoD samples. Second, it is capable of providing information for decoupling aleatoric uncertainty and epistemic uncertainty in predictions.

### Improving reliability in high-confidence prediction region for ADMET tasks

ADMET prediction is a crucial problem in drug design.^55^^,^^56^^,^^57^^,^^58^^,^^59^ Predicting and evaluating the metabolic properties of candidate drugs in the early stages of the development pipeline can help reduce clinical failure rates and development costs. However, currently available labeled datasets for various metabolic properties are relatively small, typically consisting of only a few hundred to a few thousand molecules. As a result, ADMET prediction is an application scenario where DL models are prone to OF.

#### Overall performance comparison

To evaluate model performance, two categories of metrics were employed. The first category consists of commonly used metrics for evaluating the model’s classification ability, including the area under the receiver operating characteristic curve (auROC), the area under the precision-recall curve (auPRC), accuracy (ACC), and the Matthews correlation coefficient (MCC). The second category is used to evaluate the model’s calibration capacity, comprising the expected calibration error (ECE) and the Brier score (Brier). Brier primarily assesses the accuracy of probabilistic predictions, whereas ECE evaluates the reliability of these predictions.

**Figure 4:**
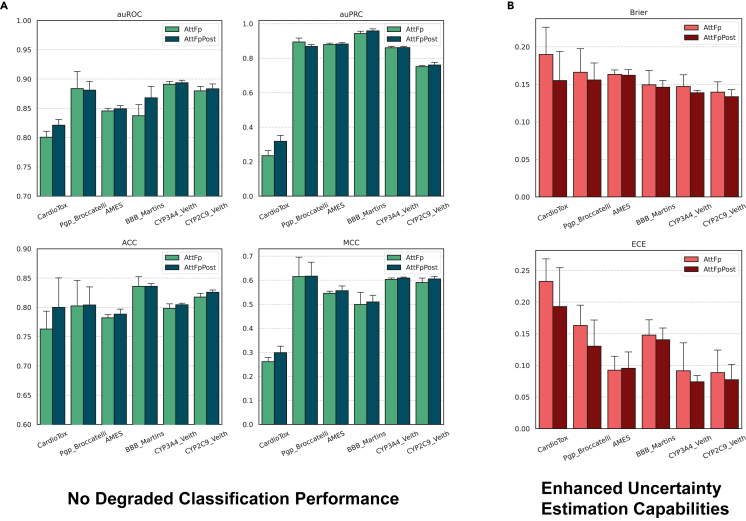
Replacing Softmax with PostNet enhances uncertainty estimation capabilities without degrading classification performance (A) Classification performance of AttFp and AttFpPost on six ADMET datasets. (B) Calibration performance of AttFp and AttFpPost on six ADMET datasets. Results are represented as mean ± SD.

Figure 4 shows the classification and calibration performance of AttFp and AttFpPost on six ADMET datasets, presented as bar graphs representing the average results of five individual models obtained through 5-fold cross-validation on the test set. As depicted in Figure 4A, it can be observed that the overall performances of AttFp and AttFpPost are comparable. An examination of the average results for four classification metrics across six ADMET datasets reveals that, of a total of 24 metrics, the average performance of the AttFpPost model using 5-fold cross-validation exceeds that of the AttFp model with the same cross-validation approach in 21 metrics, although the improvements are generally modest. These results suggest that replacing the traditional Softmax function with the PostNet structure does not impair the model’s classification performance and may even enhance it.

Figure 4B shows the calibration performance of AttFp and AttFpPost on six ADMET datasets, evaluated by ECE and Brier. It can be observed that AttFpPost outperforms AttFp in 11 of 12 metrics. This improvement is particularly prominent on the CardioTox dataset, containing a large number of OoD samples in the test set. These findings demonstrate that the modifications made to the original classification model in the form of PostNet not only enhance the reliability of the prediction but also maintain or even improve the model’s classification performance.

#### Reducing risks of misprediction in decision making

In the introduction, we hypothesize that one advantage of using PostNet is the ability to achieve stable prediction behavior for OoD samples. Due to the ambiguous criteria for defining OoD samples in datasets used for real-world molecular property prediction, our primary focus is on the model’s performance in high-confidence regions. To validate this, we used an OF rate (OFR) to indicate the model’s incorrect prediction rate in high-confidence regions ($p^{\left(i\right)}<0.1$ or $p^{\left(i\right)}>0.9$). Table 1 presents the OFR of each model based on 5-fold cross-validation on each dataset. Comparing the average results of the 5-fold models, AttFpPost exhibits a lower OFR than AttFp across all datasets. Furthermore, significance testing shows that AttFpPost exhibits a significant decrease in OFR compared with AttFp on the Pgp_Broccatelli datasets.

**Table 1:** Comparison of OFR between AttFp and AttFpPost

Dataset	AttFp OFR (↓)	AttFpPost OFR (↓)	*p* value
CardioTox	0.164 ± 0.056	0.128 ± 0.048	0.135
Pgp_Broccatelli	0.152 ± 0.034	0.110 ± 0.043∗	0.034
AMES	0.126 ± 0.012	0.124 ± 0.013	0.409
BBB_Martins	0.139 ± 0.024	0.133 ± 0.020	0.314
CYP3A4_Veith	0.086 ± 0.043	0.077 ± 0.013	0.377
CYP2C9_Veith	0.097 ± 0.030	0.089 ± 0.024	0.241

Results are represented as the mean ± SD.

∗The lower OFR with significant differences is indicated. The t test was used to obtain the *p* value.

**Figure 5:**
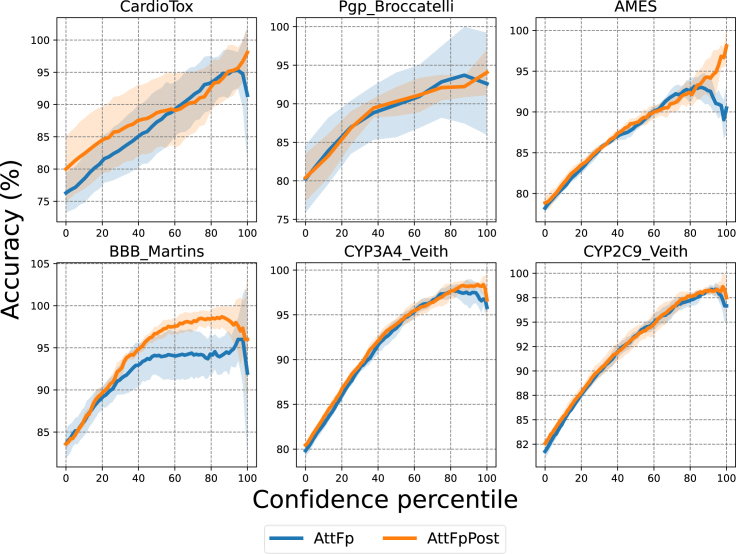
Comparison of accuracy between AttFp and AttFpPost as the uncertainty of the overall prediction results gradually decreases The confidence percentile is increased by removing prediction results with greater uncertainty. The line represents the mean accuracy, and the shaded area indicates the standard deviation.

In addition, another piece of evidence demonstrates that AttFpPost exhibits more robust uncertainty estimation in high-confidence regions compared with AttFp. Here, we use probability entropy as a measure of uncertainty in predictions, where higher entropy indicates greater uncertainty.^32^^,^^60^ Following the approach by Zhang and Lee,^9^ we ranked predictive results based on probability entropy. We then progressively removed the top *n*% of predictions with greater uncertainty to increase the confidence percentile and calculated the accuracy of the remaining predictions. Figure 5 illustrates that as predictions with greater uncertainty are gradually removed, both AttFpPost and AttFp exhibited a steady increase in accuracy for the remaining predictions. This is consistent with the expectation that the lower the uncertainty, the higher the prediction accuracy. Comparing the accuracy in high-confidence regions, AttFpPost consistently outperforms AttFp. Furthermore, on the CardioTox and AMES datasets, when only the predictions with lower uncertainty remain, the accuracy of AttFp experiences drastic fluctuations, whereas AttFpPost maintains a consistently increasing accuracy.

In our experiments on the synthetic dataset, we observed that aleatoric uncertainty and epistemic uncertainty can be decoupled based on $\alpha_{0}$ and $\alpha_{1}$. As $\alpha_{0}$ and $\alpha_{1}$ represent the model’s confidence in predicting negative and positive samples, respectively, they are influenced by the density of training samples in the corresponding latent space. Therefore, a straightforward idea is to consider the value of $\alpha_{0}+\alpha_{1}$ as the model’s epistemic uncertainty for a given prediction. Figure S1 and Table S3 show that the magnitude of $\alpha_{0}+\alpha_{1}$ exhibits a modest positive correlation with the model’s prediction accuracy. These findings support the notion that the magnitude of $\alpha_{0}+\alpha_{1}$ may be considered as a measure of the epistemic uncertainty in the prediction results, providing valuable auxiliary information for the decision-making process. However, it should be noted that determining a standard confidence interval threshold based on the magnitude of $\alpha_{0}+\alpha_{1}$ is challenging and requires further research.

Considering real-world application scenarios, there is a preference for selecting predictions with high confidence. We believe these results can substantiate that, compared with AttFp, AttFpPost can effectively reduce the risks and losses associated with mispredictions when providing auxiliary information for the decision-making process.

### Evaluating PostNet with other uncertainty estimation methods

The preceding results demonstrate the enhancement of AttFpPost over AttFp when integrated with the PostNet structure. In this section, we will explore the comparison of PostNet with other uncertainty estimation methods based on the previously used six ADMET datasets. The Ensemble^30^ and Gaussian process^61^ (GP) methods have been selected for this analysis. The Ensemble method in classification leverages the collective power of multiple models to improve prediction accuracy. It assesses uncertainty based on the agreement or variance among the predictions of these models, thereby offering a robust estimation by harnessing diverse perspectives. The GP method employs a probabilistic approach. It treats data as samples from a Gaussian distribution, providing not only class predictions but also confidence levels for these predictions.

#### Integrating PostNet and Ensemble further enhanced model performance

While the Ensemble method often requires multiple training runs to generate multiple models, thus making computational challenges, PostNet offers a viable solution to improve prediction reliability within a single model framework, potentially reducing computational demands. Importantly, PostNet can be complementary rather than in opposition to the Ensemble method. Specifically, by averaging the AttFpPost predictions of 5-fold models on each ADMET dataset, we obtained AttFpPost Ensemble. Figure S2 demonstrates that the integration of PostNet with the Ensemble method can enhance both classification and calibration performance beyond what is achievable with a single model or a simple ensemble.

#### Analogous improvements compared with Gaussian process

Han et al. proposed a GP-based GNN framework, in which the Softmax function is replaced with a differentiable GP.^29^ Here, we compared the performance of AttFpPost and AttFpGP on the CardioTox dataset, and the results are presented in Table 2. The reported mean and standard deviation of the metrics are based on five repeated experiments with the same data split strategy, using the same calculation methods as Han et al.

**Table 2:** Comparison between AttFpPost and AttFpGP based on the CardioTox dataset

Model	auROC (↑)	ECE (↓)	Brier (↓)	OFN (↓)
AttFp	0.801 ± 0.010	0.233 ± 0.036	0.190 ± 0.036	0.019 ± 0.006
AttFpGP	0.811 ± 0.013	0.202 ± 0.013	0.148 ± 0.010	0.016 ± 0.005
AttFpPost	0.821 ± 0.010	0.193 ± 0.061	0.155 ± 0.039	0.016 ± 0.004

Overconfident negative (OFN) considers only the incorrect predictions with $p^{\left(i\right)}<0.1$. This choice is made to maintain consistency with the computation method used in the study by Han et al.^29^ Results are represented as the mean ± SD.

Based on the results presented in Table 2, both AttFpGP using the GP and AttFpPost using normalizing flow outperform AttFp based on the Softmax function. AttFpGP and AttFpPost exhibit very similar performances when compared, with AttFpPost showing a slight improvement over AttFpGP in terms of auROC. This indicates that PostNet exhibits similar improvements in the ability to rank OoD samples compared with GP.

The TDC database not only provides datasets but also includes baseline models for performance comparison, such as the AttFp model with default parameters (AttFp-TDC). Table 3 presents the comparison between the main performance metrics of AttFp-TDC and our test results. Comparing AttFp-TDC and AttFpGP with AttFpPost reveals that AttFpPost outperforms AttFpGP and AttFp-TDC (except for Pgp_Broccatelli) regarding the main metrics provided by the TDC. These results also demonstrate that AttFpPost exhibits better classification performance on the specific metrics of interest to us. However, as shown in Table S4, when considering ECE and Brier, the calibration capabilities of AttFpPost appear to be slightly less optimal than those of AttFpGP.

**Table 3:** Comparison of baseline metrics reported by TDC and metrics obtained in this test

Dataset	Metric	AttFp-TDC	AttFpGP	AttFpPost
Pgp_Broccatelli	auROC	0.892 ± 0.012∗	0.876 ± 0.022	0.881 ± 0.015
AMES	auROC	0.814 ± 0.008	0.842 ± 0.005	0.849 ± 0.006∗
BBB_Martins	auROC	0.855 ± 0.011	0.857 ± 0.013	0.868 ± 0.020∗
CYP3A4_Veith	auPRC	0.851 ± 0.006	0.858 ± 0.010	0.862 ± 0.006∗
CYP2C9_Veith	auPRC	0.749 ± 0.004	0.745 ± 0.008	0.761 ± 0.016∗

Results are represented as mean ± SD.

∗The best metrics are indicated.

**Figure 6:**
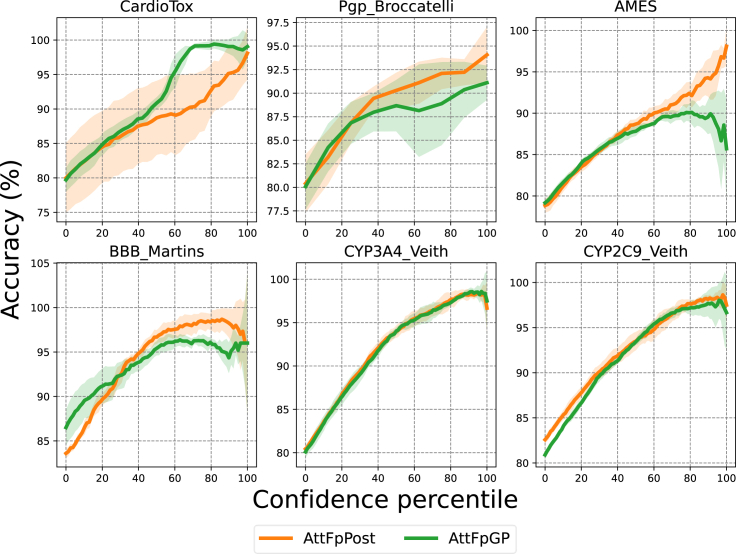
Comparison of accuracy between AttFpGP and AttFpPost as the uncertainty of the overall prediction results gradually decreases The confidence percentile is increased by removing prediction results with greater uncertainty. The line represents the mean accuracy, and the shaded area indicates the standard deviation.

Similar to Figure 5, Figure 6 illustrates a comparison of the accuracy between AttFpGP and AttFpPost in high-confidence regions. It can be observed that both AttFpGP and AttFpPost demonstrate varying levels of accuracy in high-confidence regions. Notably, on the AMES dataset, AttFpGP exhibits drastic fluctuations in accuracy within the high-confidence regions, whereas AttFpPost maintains a consistently increasing accuracy. This result suggests that, compared with the GP, PostNet may provide more valuable auxiliary decision-making information in high-confidence regions.

### Application studies

Thus far, we have demonstrated that, compared with the vanilla model, AttFpPost can achieve superior uncertainty estimation capabilities without compromising model classification accuracy. Moreover, it effectively enhances the predictive accuracy within high-confidence prediction intervals. Next, we will illustrate the utility of AttFpPost through two practical application scenarios in the context of drug design.

#### Application case 1: P-gp inhibitor classification model based on AttFpPost

In this section, we choose the classification of P-gp inhibitors as a case study to explore the contributions of AttFpPost in authentic drug discovery scenarios. P-gp, a member of the ATP-binding cassette transporter superfamily, functions as an ATP-dependent efflux pump responsible for the extrusion of drugs from cells and plays an important role in contributing to drug resistance in chemotherapy.^62^ Hence, the identification of molecules that possess the potential to inhibit P-gp in the early stages of drug discovery holds significant importance.

Currently, nearly all machine learning (ML) or DL models^63^^,^^64^^,^^65^^,^^66^^,^^67^ reported for P-gp inhibitor classification are built based on the integration of the two datasets collected by Broccatelli et al.^45^ and Chen et al.^68^ To make a fair comparison with the models reported in the literature, we trained our models using the same dataset. Specifically, we used the dataset provided by PgpRules.^63^ This dataset consists of P-gp inhibitory activity data for 2,056 molecules, including 1,244 positive instances (inhibitors) and 812 negative instances (non-inhibitors). Compared with the training set, the lack of an external test set for fair evaluation of different methods is a major challenge we currently face. Existing works either report cross-validation metrics directly on the entire dataset^63^ or perform dataset splitting based on certain conditions, such as molecular scaffolds, before reporting metrics on the test set. The inconsistency in testing methodologies and dataset splits results in incomparable performance values across different research. Therefore, we collected additional P-gp inhibitor data from the PharmaPendium database to construct a high-quality external test set, ensuring a fair comparison.

**Figure 7:**
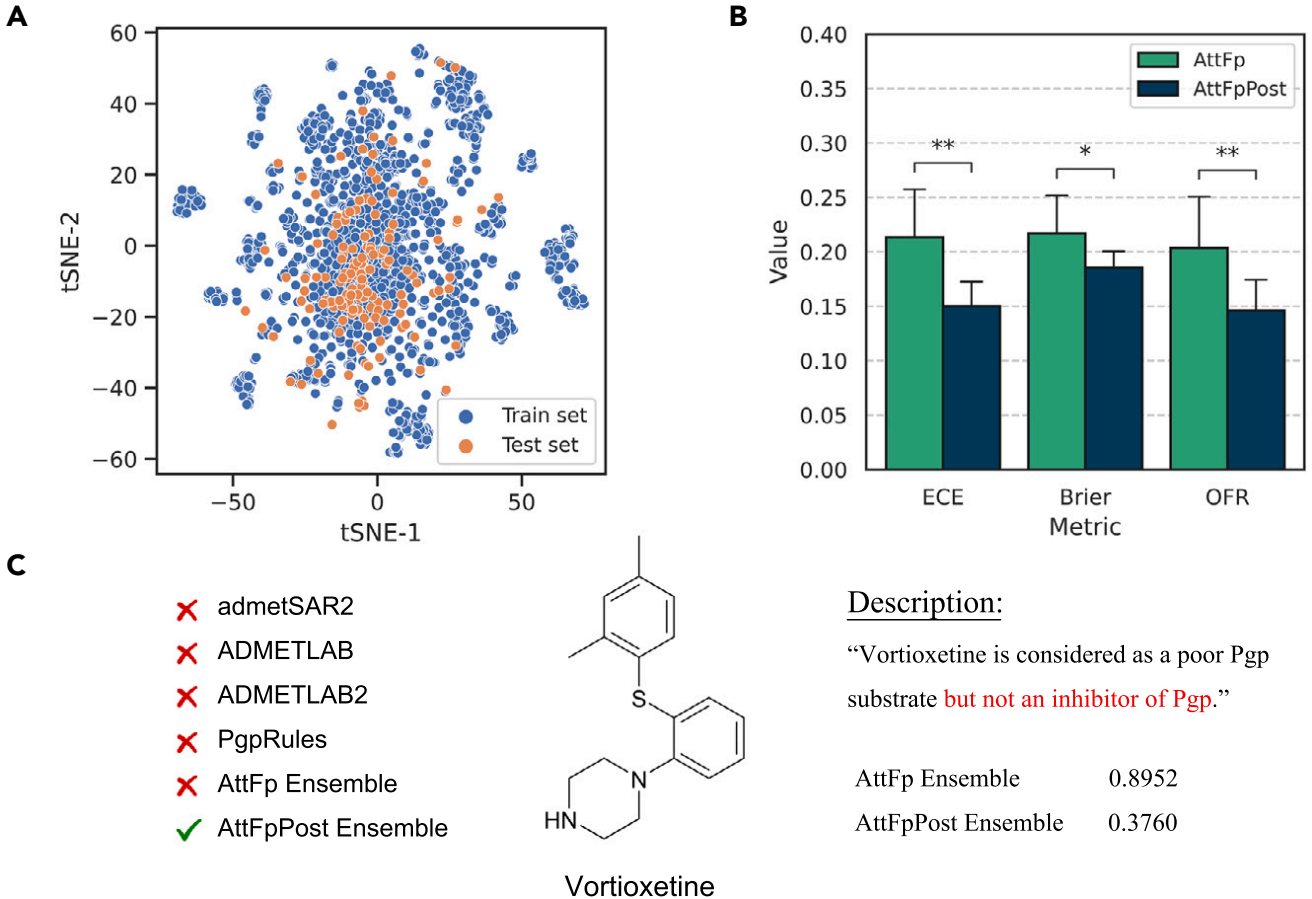
Construction of AttFpPost-based P-gp inhibitor classification model (A) t-SNE of ECFP4 molecular fingerprint for the training and external test sets. (B) ECE, Brier, and OFR values for the AttFp and AttFpPost models on the external test set. Each histogram with an error bar represents the mean and SD obtained from a 10-fold cross-validation. Statistical significance between the models was obtained using t tests, with results marked accordingly to denote levels of significance (∗*p* < 0.05, ∗∗*p* < 0.01). (C) Analysis of the prediction results for vortioxetine.

We first analyzed the predictability of the external test set from a chemical space perspective. We applied t-SNE to visualize the distribution of the ECFP4 molecular fingerprints for both the training and the external test sets. The results depicted in Figure 7A reveal that the training and external test sets cover similar chemical space ranges. However, there are some clusters of molecules in both the training and the test sets that are located outside this range, indicating low overlap between the two sets. Therefore, based on the distribution in chemical space, most of the molecules in the external test set can be considered within the application domain and can be predicted reasonably well by the model, while there may be some molecules that exhibit higher uncertainty.

We evaluated the performance of four online servers on the external test set. admetSAR2,^65^ ADMETLAB,^66^ and ADMETLAB2^67^ are widely used ADMET property prediction servers that provide predictions for whether a molecule is a P-gp inhibitor. PgpRules^63^ is a server specifically designed to predict whether a molecule is a P-gp inhibitor or substrate. The performance of four online servers is shown in Table 4. It is evident that the tested online servers exhibit varying levels of predictive capability on the external test set. In terms of the comprehensive classification metric MCC, the performance of the four servers can be ranked as follows: ADMETLAB < admetSAR2 < ADMETLAB2 < PgpRules.

**Table 4:** Classification performance of models on the P-gp inhibitor external test set

Model	ACC	Precision	Recall	F1	MCC
admetSAR2; Yang et al. ^65^	0.697	0.588	0.855	0.697	0.444
ADMETLAB; Dong et al. ^66^	0.618	0.519	0.871	0.651	0.333
ADMETLAB2; Xiong et al. ^67^	0.763	0.691	0.758	0.723	0.519
PgpRules; Wang et al. ^63^	0.756	0.658	0.839	0.737	0.530
AttFp	0.743 ± 0.035	0.668 ± 0.048	0.745 ± 0.070	0.702 ± 0.043	0.482 ± 0.071
AttFpPost	0.753 ± 0.027	0.675 ± 0.051	0.789 ± 0.084	0.722 ± 0.030	0.515 ± 0.044
AttFp Ensemble	0.776	0.689	0.823	0.750	0.558
AttFpPost Ensemble	0.796∗	0.701∗	0.871∗	0.777∗	0.605∗

The results of AttFp and AttFpPost are represented as the mean ± SD.

∗The highest value within the same metric is indicated.

Next, we evaluated the performance of AttFp and AttFpPost. We reported two sets of results for both AttFp and AttFpPost. The first set of results represents the mean and standard deviation of various metrics obtained from the 10-fold models on the external test set. The second set of results represents the metrics obtained from averaging the predictions of 10-fold models (referred to as Ensemble) on the external test set. The results are presented in Table 4.

From Table 4, it is evident that the performance of AttFpPost on the external test set outperforms AttFp across all classification metrics with the 10-fold cross-validation setting. By further averaging the predictions of the 10-fold models, the predictive performance is further improved. For example, the MCC of AttFpPost increases from an average of 0.515 to 0.605 after Ensemble, and the accuracy increases from an average of 0.753 to 0.796. AttFpPost maintains its advantage over AttFp after Ensemble, and it achieves the best performance across all metrics among all models. For instance, the MCC of AttFpPost Ensemble reaches 0.605, which is much higher than the best-performing PgpRules among the four benchmark servers (0.530).

Figure 7B presents the results of significance tests that highlight the AttFpPost model’s enhanced uncertainty estimation capabilities over the AttFp model, as evidenced by reduced ECE and Briers. These results suggest that PostNet is particularly effective in providing more reliable uncertainty estimates. In addition, AttFpPost has demonstrated a lower OFR than AttFp, further underscoring PostNet’s enhanced robustness in the high-confidence prediction region.

To further illustrate how PostNet alleviates the tendency of traditional classification models to be “overconfident,” we present an example using a representative sample, vortioxetine, from the external test set. Vortioxetine is an antidepressant drug developed through a collaboration between Lundbeck and Takeda.^69^ The chemical structure of vortioxetine is depicted in Figure 7C. The description in FDA approval documents (Figure 7C) and related research^70^ indicate that vortioxetine does not have any interaction with P-gp and does not possess P-gp inhibitory activity. However, it is worth noting that all four evaluated online servers consistently classified vortioxetine as an inhibitor, and notably, even AttFp Ensemble assigned it a positive label with a high confidence level (p=0.8952). This makes AttFpPost Ensemble the only model that correctly classifies this sample with a high uncertainty level (p=0.3760), which falls into the “weak negative” class. A structure similarity search was performed on the training set against vortioxetine, revealing the nearest sample with a Tanimoto similarity coefficient of merely 0.375. This result suggests that vortioxetine may be an OoD sample for these models. AttFpPost Ensemble successfully assigned high uncertainty for this sample, while other models erroneously provided overconfident predictions.

#### Application case 2: Improved early enrichment capability in LBVS

LBVS represents a critical approach in drug discovery for identifying lead compounds from extensive compound libraries.^71^ Once positive and negative compounds for a specific target have been acquired through a defined pathway, these data can be employed to construct a predictive model for assessing the activity of new molecules against that target. Due to the limited capacity to experimentally validate a small fraction of top-scoring molecules in each virtual screening iteration, it is of paramount importance to prevent the introduction of unreliable prediction results into the front-end enrichment process.

In this study, we conducted a comparative analysis of the performance disparities between AttFp and AttFpPost when employed for LBVS on the LIT-PCBA dataset. The experimental results are presented in Table 5. As a baseline reference, we also include the top 1% enrichment factor (EF_1%_) values for the docking software Glide and the XGBoost model based on molecular descriptors from the study conducted by Shen et al. on LIT-PCBA.^52^

**Table 5:** EF_1%_ of different models on the LIT-PCBA dataset

Target	Glide	XGBoost	AttFp	AttFpPost	*p* value
ALDH1	1.986	9.847	10.150 ± 0.451	10.345 ± 0.455∗	<0.05
FEN1	9.793	29.528	41.080 ± 1.570	41.747 ± 2.861∗	ns
GBA	2.338	14.735	22.919 ± 2.486	28.771 ± 4.973∗	<0.05
KAT2A	2.120	11.077	12.916 ± 2.041∗	10.416 ± 1.863	ns
MAPK1	3.852	9.049	5.190 ± 1.835	7.267 ± 1.942∗	ns
PKM2	2.810	7.471	6.902 ± 0.996	7.490 ± 1.263∗	ns
VDR	0.656	10.067	15.838 ± 1.952	16.617 ± 1.673∗	ns

The results of AttFp and AttFpPost are represented as the mean ± SD. Statistical t tests were applied between AttFp and AttFpPost, with “<0.05” denoting statistical significance and “ns” denoting non-significance.

∗The AttFp and the corresponding AttFpPost with higher mean EF_1%_ values are indicated. The model with the highest mean EF_1%_ value among all models is indicated with an underline.

Table 5 presents the experimental results on the LIT-PCBA dataset. It is evident that the screening power of the GNN is superior to that of molecular docking software Glide and classical ML models such as XGBoost. Jiang et al. recently compared various GNN models and ML models on 11 public datasets and concluded that ML models based on descriptors outperform GNN models in molecular property prediction.^72^ However, there is currently no study comparing these two models in LBVS using large-scale virtual screening datasets. From Table 5, it can be observed that GNN outperforms XGBoost in terms of screening power across almost all targets (except MAPK1). In certain targets, such as KAT2A and PKM2, XGBoost performs similar to the GNN or even surpasses some GNN models. But overall, it does not achieve the best performance. For certain targets such as GBA, the benefit of using the GNN is particularly pronounced. These results indicate that, although training the GNN on datasets with a high number of samples such as LIT-PCBA can be computationally intensive and time consuming, especially compared with ML models, it still offers many enrichment benefits overall. Therefore, it can be considered a viable option when the resources and conditions allow for it.

In Table 5, the higher mean EF_1%_ of AttFpPost over AttFp is indicated by an asterisk. It is evident that AttFpPost with the PostNet structure achieved higher EF_1%_ in most targets, except for KAT2A. Notably, significance tests reveal that AttFpPost significantly outperforms AttFp in targets such as ALDH1 and GBA.

Previous studies have demonstrated that BNNs have stronger screening power than traditional classification models in LBVS.^13^ We believe that PostNet may also follow the same principles. However, PostNet uses normalizing flow to fit the latent representation distributions of positive and negative molecules, which allows it to capture more information compared with traditional classification models. This could be another reason the PostNet exhibits stronger screening capabilities.

## Discussion

This work explored the application of an EDL model, namely PostNet, in the context of molecular property prediction for classification tasks. We highlighted the limitations of traditional classification models that use the Softmax function, with particular emphasis on the potential risk of giving OF predictions for OoD samples, which can be particularly problematic when using computational models for practical applications, especially in drug development. Furthermore, we introduced the basic principles of PostNet and analyzed why it can overcome certain problems in terms of model structure. To prove that PostNet can decouple the total uncertainty, we first constructed a synthetic dataset that simulated the idealized condition of high aleatoric uncertainty and high epistemic uncertainty. The results demonstrated that AttFpPost with the PostNet architecture can effectively distinguish samples with high epistemic uncertainty and exclude them from the high-confidence region. We further examined the model performance in real-world application scenarios based on six ADMET properties prediction tasks. The results demonstrated that PostNet can reduce OF predictions while maintaining or even enhancing classification performance compared with traditional classification models. We then constructed a P-gp inhibitor classification model based on AttFpPost. The results on an external test set showed that the model exhibited better classification capability compared with the existing ADMET prediction servers. Finally, on the LIT-PCBA dataset, we demonstrated that PostNet also exhibited better performance in LBVS.

The reliable AI remains central to the future direction of AIDD. Although our study underscores the potential of PostNet in reducing OF predictions, there remains a substantial amount of progress to be made within the realm of AIDD. Our model has demonstrated the ability to identify OoD samples in synthetic experiments and will further deepen the research on identifying OoD molecules in real application scenarios in the future. Another immediate challenge to address is the development of an appropriate evaluation framework for uncertainty estimation methods, tailored to the unique context of drug development scenarios. In addition, the continuous refinement of predictive models to enhance accuracy, robustness, and interpretability stands as an ongoing objective.

## Experimental procedures

### Resource availability

#### Lead contact

Requests for further information and resources and reagents should be directed to and will be fulfilled by the lead contact, Dingyan Wang (wangdy@lglab.ac.cn).

#### Materials availability

This study did not generate new unique materials.

#### Data and code availability

The data and code used to generate the results shown in this study are available at GitHub (https://github.com/HShokaku/AttFpPost) and Zenodo.^73^

### Methods

#### Normalizing flow

One of the key elements to ensure the prediction accuracy of PostNet is the design of $P_{c}\left(z\right)$. $P_{c}\left(z\right)$ needs to meet two requirements: (1) strong fitting ability and (2) convenience for training and updating. To satisfy these criteria, PostNet used normalizing flow^74^ to ensure that $P_{c}\left(z\right)$ can effectively fit the corresponding probability density functions over Dirichlet parameters in latent space. Normalizing flow is a class of generative models used to model complex data distributions by transforming a simple distribution (typically a Gaussian) into a complex data distribution through a series of invertible neural network layers. Based on the properties of normalizing flow, it can conveniently and accurately estimate the probability density of samples within the real data distribution through the invertible neural network layers.^75^ According to previous studies, normalizing flow is theoretically capable of modeling any continuous distribution given an expressive and deep enough model.^74^^,^^76^

#### Loss function

As mentioned in the fundamentals of PostNet, PostNet predicts the parameters of $q^{\left(i\right)}$ to obtain the posterior of the categorical distribution $p^{\left(i\right)}$. When optimizing $q^{\left(i\right)}$ during the training process, $q^{\left(i\right)}$ should both explain the training data well and penalize deviations from the smooth prior distribution. This is similar to the form of evidence lower bound loss function commonly used in variational inference. Therefore, the designed loss function consists of two components, $E_{q\left(p^{\left(i\right)}\right)}\left[\text{CE}\left(p^{\left(i\right)},y^{\left(i\right)}\right)\right]$ and $H\left(q^{\left(i\right)}\right)$, as shown in Equation 7:

(Equation 7) $$\begin{array}{c} L\left(\theta ,\varphi \right)=\frac{1}{N}\sum_{i=1}^{N}\left(E_{q\left(p^{\left(i\right)}\right)}\left[\text{CE}\left(p^{\left(i\right)},y^{\left(i\right)}\right)\right]-H\left(q^{\left(i\right)}\right)\right)\text{,} \end{array}$$

where the first term $E_{q\left(p^{\left(i\right)}\right)}\left[\text{CE}\left(p^{\left(i\right)},y^{\left(i\right)}\right)\right]$ corresponds to the uncertain cross-entropy loss, which is used to increase the confidence for observed data.^77^ CE is the cross-entropy loss function used in the general classification model. The second term $H\left(q^{\left(i\right)}\right)$ is an entropy regularizer to smooth distributions $q^{\left(i\right)}$. H represents the entropy of the probability distribution. θ and φ are the parameters of the model for feature extraction and the parameters of the normalizing flow (related to the calculation of $p^{\left(i\right)}$), respectively. N is the number of training data.

Considering that $q^{\left(i\right)}$ obeys the Dirichlet distribution, the first term can be calculated using the following equation:

(Equation 8) $$\begin{array}{c} E_{q\left(p^{\left(i\right)}\right)}\left[\text{CE}\left(p^{\left(i\right)},y^{\left(i\right)}\right)\right]=\Psi \left(\alpha_{0}^{\left(i\right)}+\alpha_{1}^{\left(i\right)}\right)-\Psi \left(\alpha_{c^{*}}^{\left(i\right)}\right)\text{,} \end{array}$$

where Ψ is the digamma function and $c^{*}$ is the true label (0 for negative and 1 for positive).

### Evaluation metrics

The evaluation of accuracy includes the commonly used metrics auROC, auPRC, ACC, Precision, Recall, F1, and MCC. Calibration evaluation was conducted using the common metrics ECE and Brier. The calculation of ECE and Brier is consistent with the source code of Han et al.^29^ To investigate whether PostNet can reduce the percentage of false high-confidence predictions compared with traditional classification models, we followed Han et al.^29^ to define “high-confidence predictions” as samples with $p^{\left(i\right)}<0.1$ or $p^{\left(i\right)}>0.9$. We further defined the OFR as the percentage of samples in high-confidence predictions with $p^{\left(i\right)}<0.1$ and $y^{\left(i\right)}=1$ or $p^{\left(i\right)}>0.9$ and $y^{\left(i\right)}=0$.

In virtual screening experiments, EF_1%_ was used to evaluate the screening power of the model, and the BEDROC (Boltzmann-enhanced discrimination of receiver operating characteristic)^78^ was used as the early stopping criterion for model training. The calculation of EF_1%_ and BEDROC was consistent with the source code of Shen et al.^52^

The details of the metrics calculation are explained in the supplemental experimental procedures.

### Training details

#### Additional hyperparameters of AttFpPost

Due to the replacement of the Softmax function with the normalizing flow, AttFpPost has two additional tunable hyperparameters compared with AttFp: the number of invertible mappings (*n_density*) and the dimensionality of the latent space (*latent_dim*). These hyperparameters play a crucial role in PostNet’s architecture.^37^ A grid search was performed on these two hyperparameters using the Pgp_Broccatelli dataset to preliminarily determine a reasonable range. When the values of the two hyperparameters are within the range of 10, the performance of the model exhibits small fluctuations, as shown in Figure S3. This is consistent with the findings of Charpentier et al.^37^ Therefore, in subsequent experiments, we set the search range for *latent_dim* and *n_density* to be from 2 to 10.

#### Hyperparameter optimization

We adopted distinct training strategies tailored to different tasks. For tasks requiring hyperparameter optimization, we employed Bayesian optimization^79^^,^^80^ to explore the hyperparameter space.

#### Model ensemble

We conducted cross-validation or parallel training of multiple models under the same set of hyperparameters to obtain an Ensemble model for each task.

The Bayesian hyperparameter optimization space of AttFp and AttFpPost is listed in Table S5. The grid search space for determining *n_density* and *latent_dim* and the training strategies tailored to different datasets are detailed in the supplemental experimental procedures.

### Implementation

The foundational model of AttFp is built upon DGL-LifeSci^81^ and Chemprop.^82^ The normalizing flow used in the output layer of PostNet is built upon Pyro.^83^ The GP is implemented based on GPyTorch.^84^ Model training is based on the PyTorch^85^ DL framework.

## Supplemental information

Document S1. Figures S1–S3 and Tables S1–S5

Document S2. Article plus supplemental information
