# Supplementary material for: Reducing overconfident errors in molecular property classification using Posterior Network
Source: Patterns (N Y). 2024 May 8;5(6):100991. doi: 10.1016/j.patter.2024.100991 (PMC11240180; doi:10.1016/j.patter.2024.100991)
Supplement: Document S1. Figures S1–S3 and Tables S1–S5 [file mmc1.pdf]

Patterns, Volume *5*

Supplemental information

Reducing overconfident errors in molecular property classification using Posterior Network

Zhehuan Fan, Jie Yu, Xiang Zhang, Yijie Chen, Shihui Sun, Yuanyuan Zhang, Mingan Chen, Fu Xiao, Wenyong Wu, Xutong Li, Mingyue Zheng, Xiaomin Luo, and Dingyan Wang

1 Data profile

Table S1 Information of datasets used in the ADMET prediction test

Dataset	Total Data Size	Numbers of Positive Samples	Numbers of Negative Samples	Modeling Task & Dataset Source
CardioTox	8981	2215	6766	Whether to inhibit hERG
Pgp_Broccatelli	1219	651	568	Whether to inhibit P-gp
AMES	7278	3974	3304	Whether to induce genetic damage and frameshift mutations
BBB_Martins	2039	1560	479	Whether it can cross the blood-brain barrier
CYP3A4_Veith	12328	5110	7218	Whether to inhibit CYP3A4
CYP2C9_Veith	12092	4045	8047	Whether to inhibit CYP2C9

Table S2 Sizes and data distribution of each target in LIT-PCBA dataset

Target name	Training set		Test set		Ratio
	Number of positive molecules	Number of negative molecules	Number of positive molecules	Number of negative molecules	
ALDH1	4016	76384	1334	25666	~1:19
FEN1	269	263257	90	88344	~1:979
GBA	122	218681	41	73434	~1:1792
KAT2A	145	257616	48	86548	~1:1777
MAPK1	228	46066	77	15510	~1:202
PKM2	410	182958	136	61081	~1:446
VDR	460	188967	154	63423	~1:411

2 Additional Results

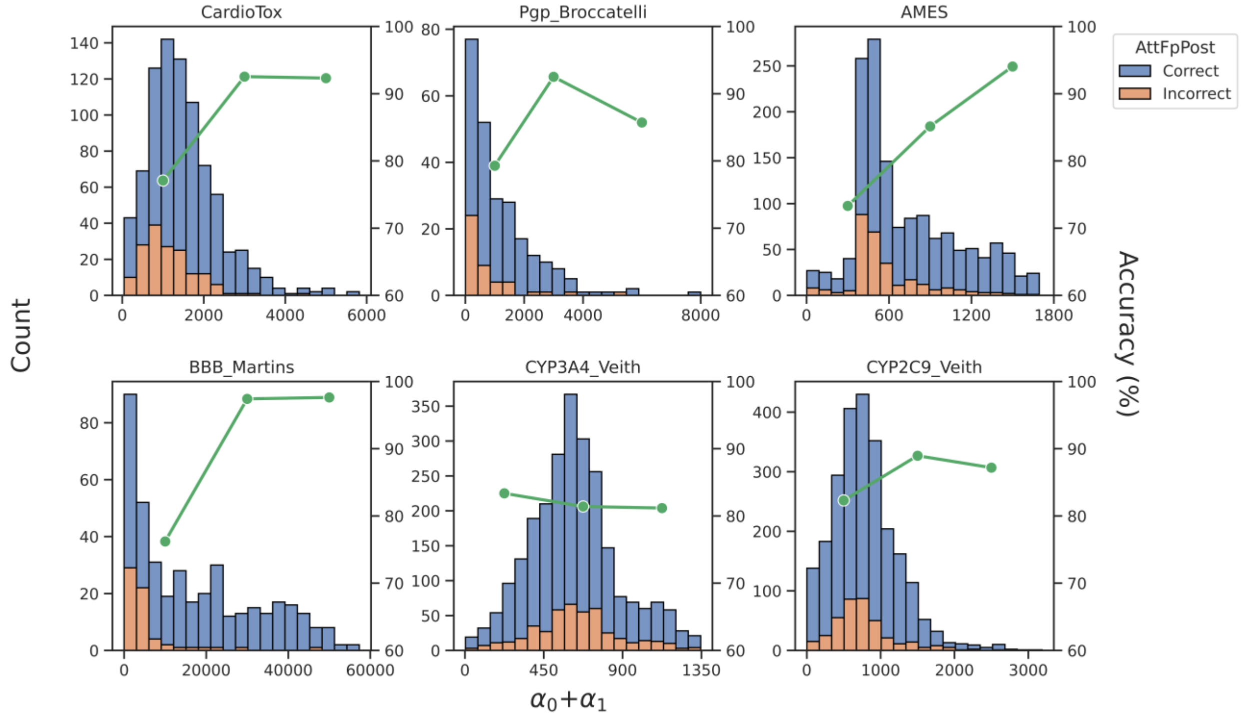

Figure S1. Distribution of prediction results and accuracy of AttFpPost on six ADMET datasets across different $\alpha_0 + \alpha_1$ intervals

The blue bars represent the count of correct predictions, the orange bars represent the count of incorrect predictions. The abscissa scale shows three intervals (high, medium, low) for epistemic uncertainty, based on the max value of $\alpha_0 + \alpha_1$ from AttFpPost. The segmented green line indicates prediction accuracy in these intervals.

Table S3 Spearman correlation analysis of AttFpPost prediction correctness and $\alpha_0 + \alpha_1$.

Dataset	Spearman Correlation Coefficient	p-value
CardioTox	0.222	8.56e-11
Pgp_Broccatelli	0.233	2.39e-04
AMES	0.205	2.51e-15
BBB_Martins	0.373	7.61e-15
CYP3A4_Veith	-0.031	0.124
CYP2C9_Veith	0.040	0.0476

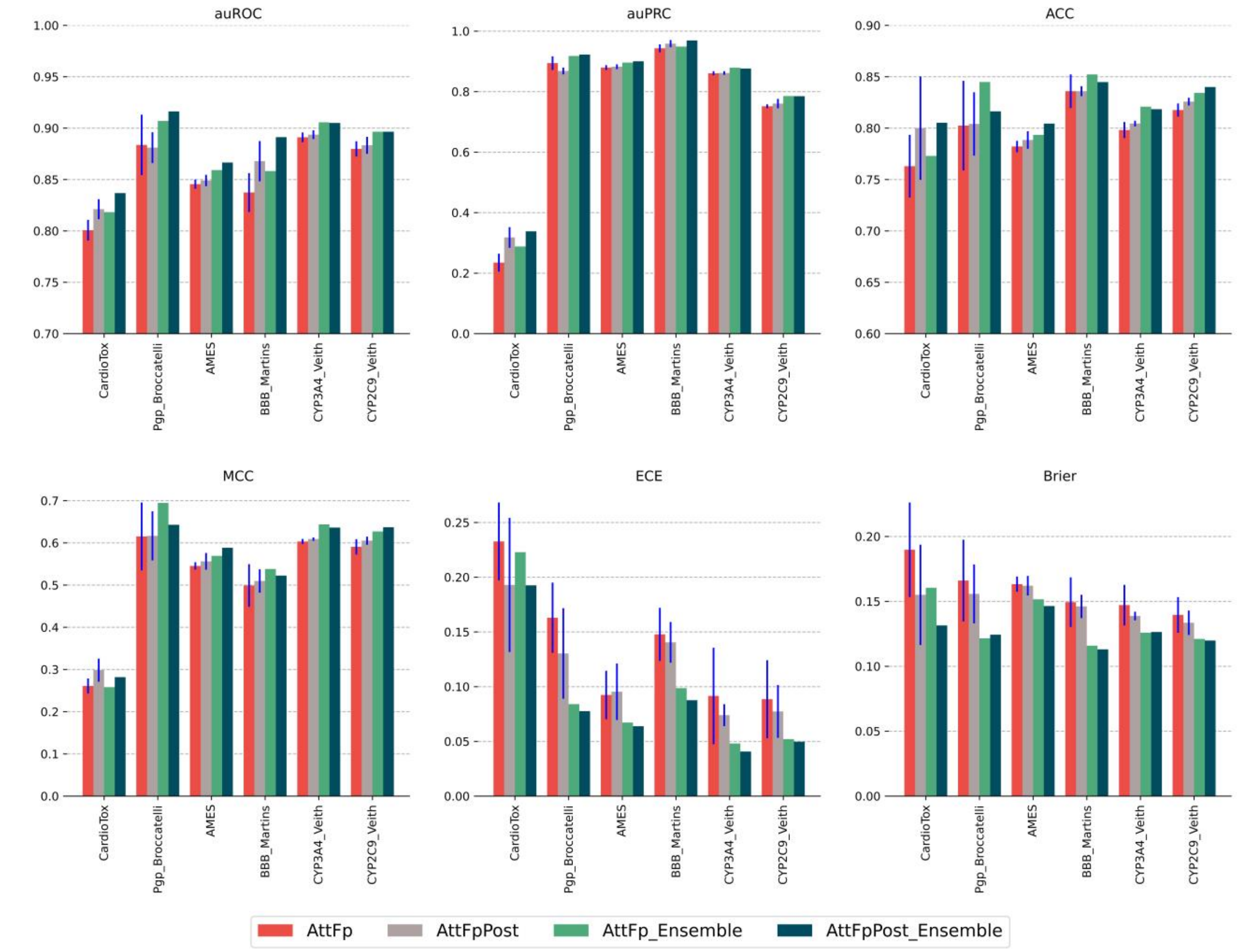

Figure S2 Integrating PostNet with Ensemble method further enhance the model performance

Results are represented as mean ± SD.

Table S4 Comparing calibration capabilities of AttFpGP and AttFpPost

Dataset	Metric	AttFpGP	AttFpPost
Pgp_Broccatelli	Brier (↓)	0.156±0.016	0.156±0.023
	ECE (↓)	0.139±0.016	0.130±0.041
AMES	Brier (↓)	0.153±0.002	0.162±0.008
	ECE (↓)	0.033±0.007	0.095±0.026
BBB_Martins	Brier (↓)	0.114±0.007	0.146±0.009
	ECE (↓)	0.107±0.015	0.140±0.019
CYP3A4_Veith	Brier (↓)	0.138±0.006	0.139±0.003
	ECE (↓)	0.062±0.018	0.074±0.010
CYP2C9_Veith	Brier (↓)	0.134±0.003	0.134±0.009
	ECE (↓)	0.045±0.014	0.077±0.024

Results are represented as mean ± SD.

3 Supplemental Experimental Procedures

a) Evaluation metrics

The evaluation of accuracy includes the commonly used metrics: auROC (area under the receiver operating characteristic curve), auPRC (area under the precision-recall curve), accuracy (ACC), Precision, Recall, F1, and Matthews correlation coefficient (MCC). The equations for the latter five metrics are:

$$\mathrm{ACC} = \frac{\mathrm{TP} + \mathrm{TN}}{\mathrm{TP} + \mathrm{FP} + \mathrm{TN} + \mathrm{FN}} \tag{1}$$

$$\mathrm{Precision} = \frac{\mathrm{TP}}{\mathrm{TP} + \mathrm{FP}} \tag{2}$$

$$\mathrm{Recall} = \frac{\mathrm{TP}}{\mathrm{TP} + \mathrm{FN}} \tag{3}$$

$$\mathrm{F1} = \frac{2 \times \mathrm{Precision} \times \mathrm{Recall}}{\mathrm{Precision} + \mathrm{Recall}} \tag{4}$$

$$\mathrm{MCC} = \frac{\mathrm{TP} \times \mathrm{TN} - \mathrm{FN} \times \mathrm{FP}}{\sqrt{(\mathrm{TP} + \mathrm{FN})(\mathrm{TP} + \mathrm{FP})(\mathrm{FP} + \mathrm{FN})(\mathrm{FN} + \mathrm{TN})}} \tag{5}$$

where TP is the number of true positives, TN is the number of true negatives, FP is the number of false positives, and FN is the number of false negatives.

The top 1% enrichment factor ($EF_{1\%}$) was used to evaluate the screening power of the model in virtual screening experiments. $EF_{1\%}$ measures the ratio of the percentage of positive molecules in the top 1% of molecules enriched by the model to the percentage of positive molecules in the test set. BEDROC (Boltzmann-enhanced discrimination of receiver operating characteristic)[1] was used as the early stopping criteria for model training in virtual screening experiments. The calculation of $EF_{1\%}$ and BEDROC was consistent with the source code of Shen et al.[2].

Calibration evaluation was done by using common metrics Expected Calibration Error (ECE) and Brier Score (Brier). The Brier can be calculated as follows:

$$\mathrm{Brier} = \frac{1}{N}\sum_{i=1}^{N}\left(p^{(i)} - y^{(i)}\right)^2 \tag{6}$$

where $p^{(i)}$ and $y^{(i)}$ are the predictive probability and the label of the i-th test sample, and N is the total number of the test samples. Brier penalizes the predicted probability for deviations from the true label, and the lower its value, the better calibration of the model. In essence, Brier actually penalizes the uncertainty of the prediction result, and the smaller Brier, the higher confidence prediction result shows.

The ECE was calculated by sorting the test set in ascending order $p^{(i)}$, and then dividing it into M intervals based on quantiles or ranges of the predicted values. Subsequently, the Fraction of Positives (FOP) and the Mean Predicted Value (MPV) of the m-th interval, denote as B_m, were defined as follows:

$$\mathrm{FOP}(B_m) = \frac{1}{|B_m|} \sum_{i \in B_m} \mathbb{1}\left(y^{(i)} = 1\right) \tag{7}$$

$$\mathrm{MPV}(B_m) = \frac{1}{|B_m|} \sum_{i \in B_m} p^{(i)} \tag{8}$$

where $|B_m|$ is the number of test samples contained in B_m. $\mathbb{1}$ is an indicator function that returns 1 if the input is true and 0, otherwise. $y^{(i)} = 1$ indicates whether the true label of the i-th test sample is positive (1) or negative (0). The ECE over the entire test set can be calculated as follows:

$$\mathrm{ECE} = \sum_{m=1}^{M} \frac{|B_m|}{N} \left|\mathrm{FOP}(B_m) - \mathrm{MPV}(B_m)\right| \tag{9}$$

where N is the total number of samples in the entire test set. The calculation of ECE and Brier is consistent with the source code of Han et al.[3]

To investigated whether PostNet can reduce the percentage of false high-confidence predictions compared to traditional classification models. We followed Han et al.[3] to defined "high-confidence predictions" as samples with $p^{(i)} < 0.1$ or $p^{(i)} > 0.9$, and further defined Overconfident False samples Rate (OFR) as the percentage of samples in high-confidence predictions with $p^{(i)} < 0.1$ and $y^{(i)} = 1$ or $p^{(i)} > 0.9$ and $y^{(i)} = 0$:

$$\mathrm{OFR} = \frac{\sum_i \mathbb{1}\left(\left(p^{(i)} < 0.1 \text{ and } y^{(i)} = 1\right) \text{ or } \left(p^{(i)} > 0.9 \text{ and } y^{(i)} = 0\right)\right)}{\sum_i \mathbb{1}\left(p^{(i)} < 0.1 \text{ or } p^{(i)} > 0.9\right)} \tag{10}$$

b) Training details

Determining a reasonable range for *n_density* and *latent_dim*

In the original literature of PostNet by Charpentier *et al*., the default settings for *n_density* and *latent_dim* were both set to 6. The authors also suggested that *latent_dim* should be set to a value close to the number of classes in the prediction tasks. They found that if *latent_dim* is too large (e.g., 32), it can lead to the "curse of dimensionality" effect, where the predictive performance and uncertainty estimation of the model both deteriorate significantly.

To preliminarily determine the reasonable range of *n_density* and *latent_dim* in AttFpPost, we performed a grid search on these two hyperparameters using the Pgp_Broccatelli dataset. We considered 5 possible values for *n_density*: 2, 4, 6, 8, and 10. For *latent_dim*, we considered 9 possible values: 2, 4, 6, 8, 10, 15, 20, 25, and 30. The remaining hyperparameters were set to their default values. For each combination, we conducted 5-fold cross-validation on the training set and recorded the mean and standard deviation of auROC. The results are shown in Figure S3.

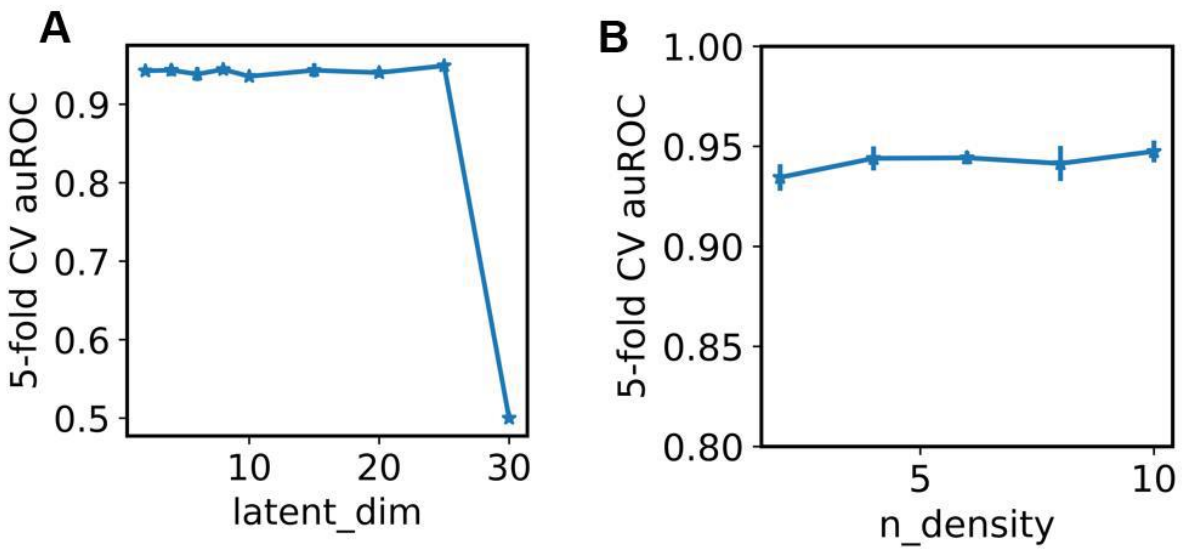

Figure S3. Relationship between the setting of parameters of normalizing flow and model performance

(A) Effect of *latent_dim* on model performance.

(B) Effect of *n_density* on model performance.

Figure S3A shows that when *latent_dim* is less than or equal to 25, the model's performance exhibits small fluctuations without significant differences. However, when *latent_dim* is set to 30, the model's performance dramatically declines, with almost no predictive capability (auROC of 0.5). This is consistent with the findings of Charpentier *et al*. Similarly, for different values of *n_density* (Figure S3B), the model's performance also shows small fluctuations without a clear increasing or decreasing trend. Taking into account the aforementioned results and the available computational resources, we set the search range for *latent_dim* and *n_density* to be from 2 to 10 in the subsequent experiments.

Hyperparameter optimization search space

Table S5 Hyperparameter searching space of AttFp and AttFpPost. If there are 3 numbers in the brackets, the parameter is a discrete variable, the first number indicates the minimum value, the second number indicates the maximum value, and the third number indicates the value interval; if there are 2 numbers in the brackets, the parameter is a continuous variable, the first number represents the minimum value, and the second number represents the maximum value.

Hyperparameter	Description	Range
hidden_size	the dimensions of hidden layers	(300, 600, 1)
radius	the number of attentive layers for atom embedding	(2, 6, 1)
T	the number of attentive layers for molecule embedding	(1, 5, 1)
p_dropout	dropout rate	(0, 0.5, 0.05)
lr	learning rate	(1e-4, 1e-2)
ffn_num_layers	the number of fully connected layers	(2, 4, 1)
n_density	the number of normalizing flow layers	(2, 10, 1)
latent_dim	the dimensions of normalizing flow	(2, 10, 1)

Training strategies tailored to different tasks

CardioTox. We followed Han et al.'s training-validation-test split method for investigating the prediction behavior of PostNet on OoD samples. Bayesian optimization was employed for both AttFp and AttFpPost to explore the hyperparameter space. Due to the fixed training-validation split of the CardioTox dataset, the hyperparameter search for this dataset is based on the average performance of the model on the validation set after 5 rounds of parallel training.

Therapeutics Data Commons database. For the five ADMET datasets provided in Therapeutics Data Commons (TDC) database, we utilized the training-test split method provided by TDC and further randomly split the training set into five folds for 5-fold cross-validation. Bayesian optimization was employed for both AttFp and AttFpPost to explore the hyperparameter space.

P-gp inhibitors classification model. We randomly split the training into 10-fold subsets, ensuring a consistent ratio of positive and negative samples in each subset. We then performed 10-fold cross-training on AttFp and AttFpPost using the optimal hyperparameters obtained from the previous Pgp_Broccatelli task.

LIT-PCBA. Given the vast number of samples available for each target in the LIT-PCBA dataset, it is not feasible to individually search hyperparameters for each target. To strike a balance between available computational resources and fairness in the evaluation process, we employed two strategies to expedite model training. Firstly, we trained the model using the default hyperparameters of AttFp. Second, during the training process, we employed a negative sampling strategy. For each epoch, we constructed a sub-training set using all positive compounds and an equal number of randomly sampled negative compounds from the training set. To prevent underfitting, we extended the patience parameter and the interval for checking early stopping. Specifically, after every 300 parameter updates, we performed testing on the validation set to

calculate the BEDROC score. If the BEDROC score did not improve for 50 consecutive tests on the validation set, we considered the model to have converged and stopped the training process. The best ensembled model on the validation set was selected as the final model for testing on the test set.

c) Detailed data collection strategy for P-gp external test set

Pharmapendium is a drug safety, efficacy and Drug Metabolism and Pharmacokinetics (DMPK) database developed by Elsevier, and its data sources include drug approval documents from the Food and Drug Administration (FDA) in the United States and the European Medicines Agency (EMA) in Europe. We only retained entries that met the following criteria: (1) the number of heavy atoms is less than 100, (2) they have clear labels (Yes or No) indicating P-gp inhibition activity, and (3) they have not appeared in the training set. Furthermore, we excluded molecules with qualitative labels that had multiple conflicting records. For the remaining molecules, we conducted a manual inspection based on the corresponding approval documents or literature provided by Pharmapendium. During the initial screening process, we accessed the "Metabolizing Enz. & Transporters data search" module in Pharmapendium. We set the search criteria as follows: "Data type" was set to "Transporter Inhibitor", "Enzyme/transporter name" was set to "MDR1", and "Species" was set to "Human".

Each label in the external test set can be traced back to a qualitative statement in the original records. For example, the drug Afatinib has a qualitative statement in its approval document that states "Afatinib is a P-gp inhibitor". By using qualitative statements to determine the labels, we can avoid the challenges associated with different measurement indicators and varying

classification thresholds under different experimental conditions. This approach is more aligned with real-world applications and further enhances the data quality.

References

1. Truchon, J.-F., and Bayly, C.I. (2007). Evaluating virtual screening methods: good and bad metrics for the "early recognition" problem. Journal of chemical information and modeling *47*, 488-508.

2. Shen, C., Weng, G., Zhang, X., Leung, E.L.-H., Yao, X., Pang, J., Chai, X., Li, D., Wang, E., and Cao, D. (2021). Accuracy or novelty: what can we gain from target-specific machine-learning-based scoring functions in virtual screening? Briefings in Bioinformatics *22,* bbaa410.

3. Han, K., Lakshminarayanan, B., and Liu, J. (2021). Reliable graph neural networks for drug discovery under distributional shift. arXiv preprint arXiv:2111.12951.
